# Comparison of the Web-Based and Digital Questionnaires of the Spanish and Catalan Versions of the KIDSCREEN-52

**DOI:** 10.1371/journal.pone.0114527

**Published:** 2014-12-05

**Authors:** Luis Rajmil, Noemí Robles, Dolors Rodriguez-Arjona, Marta Azuara, Francisco Codina, Hein Raat, Ulrike Ravens-Sieberer

**Affiliations:** 1 Agència de Qualitat i Avaluació Sanitàries de Catalunya, Barcelona, Spain; 2 IMIM (Institut Hospital del Mar de Recerca Biomèdica), Barcelona, Spain; 3 Centro de Investigación Epidemiológica en Red (CIBERESP), Madrid, Spain; 4 Corporació de Salut del Maresme i la Selva, Sant Jaume, 209–217, 08370 Calella, Spain; 5 Department of Public Health, Erasmus MC – University Medical Center Rotterdam, The Netherlands; 6 Department of Child and Adolescent Psychiatry, Psychotherapy, and Psychosomatics, University Medical Center Hamburg-Eppendorf, Martinistr. 52 (W29), 20246 Hamburg, Germany; University of New South Wales, Australia

## Abstract

**Background:**

The objectives of the study were to develop web-based Spanish and Catalan versions of the KIDSCREEN, and to compare scores and psychometric properties with the paper version.

**Methods:**

Internet and paper Spanish and Catalan versions of the KIDSCREEN-52 were included in a cross-sectional study in school-age children. Web-based and paper Spanish or Catalan versions of the KIDSCREEN-52 were administered to students aged 8 to 18 years from primary and secondary schools in Palafolls (Barcelona, Spain, n = 923). All students completed both web-based and paper versions during school time with an interval of at least 2 hours between administrations. The order of administration was randomized. The KIDSCREEN-52, the Strengths and Difficulties Questionnaire (SDQ), and sociodemographic variables were collected. Missing values, floor and ceiling effects, and internal consistency were compared between both versions, as well as mean score differences, level of agreement, and known groups and construct validity.

**Results:**

Participation rate was 77% (n = 715). Web-based and paper versions showed low percentage of missing values and similar high ceiling effect (range 0 to 44%). Mean score differences showed an effect size (ES) lower than 0.2 in all dimensions. Internal consistency ranged from 0.7 to 0.88, and degree of agreement was excellent (Intraclass correlation coefficient [ICC] range 0.75 to 0.87). Expected differences were seen by sex, age, socioeconomic status and mental health status.

**Conclusions:**

The web-based KIDSCREEN-52 showed similar scale score and reliability and validity than the paper version. It will incorporate the child population in the assessment of quality of life providing a more attractive format.

## Introduction

The use of information and communication technology (ICT) in healthcare services seems to be one of the most promising current areas of research. Innovation in the area of health in recent years is concentrated largely in ICTs [1]. Children and adolescents are population groups who could most benefit from the use of ICTs. Administration of standardized paper questionnaires of health-related quality of life (HRQOL) instruments addressed to children would be probably increasingly replaced by digital versions, specifically designed for children and teenagers [2]. This requires the development of questionnaires via web and the comparison to the original paper versions.

From the perspective of clinical practice and research the use of Internet avoids printing paper, data entry is done online, and further procedures may be employed to reduce the number of missing responses. In general, the mode of administration of the questionnaire can affect participation rate, the number of missing responses, psychometric properties, and scores [3]. Some studies showed minor differences between computer and paper versions of the same questionnaires [4], [5]. When the same questionnaire on paper and computer were compared and the contents were sensitive and/or committed such as drug use or sexual behavior, it was observed that administration via computer increased coverage because it was perceived as providing more intimacy than the paper format [2].

The KIDSCREEN is a HRQOL instrument addressed to children 8 to 18 years old developed in Europe and used worldwide [6]. Although several studies used electronic administration of the instrument, a few studies to date have been published on the psychometric properties of this version. A digital version adapted to deaf children has been developed [7]. An electronic version of the KIDSCREEN-27 has demonstrated acceptable psychometric properties without face to face comparison with the paper version [8]. However, it is widely accepted that measurement properties of any new administration form should be compared with that of the original version [9]. The objectives of the present study were to develop web-based Spanish and Catalan versions of KIDSCREEN-52 (eKIDSCREEN), and to compare scores and psychometric properties with the paper version.

## Methods

From the conventional paper format, using the same wording of the items and instructions, an internet version of the questionnaire was developed through a generic internet tool using Ruby on Rails applications and MySQL database (http://rubyonrails.org). The design of the web version was done trying to present a visual format to participants as similar as possible to the paper version. The internet version did not allow the respondent to select more than one answer to each item and it checked the questionnaire for missing answers before the respondent could “logout”. A pilot test was carry out including approximately 20 simulations to detect inconsistencies, errors and problems with the use of the digital version.

### Sample selection and procedures

All students were selected from 3rd to 6th course of Primary education (approximately 8 to 11 years old), 1st through 4th grade of Secondary education (12–16y), and High School (17–18y) of all 3 schools in Palafolls, a village from Barcelona province with approximately 9000 inhabitants (n = 923). All students completed both paper and digital versions during the same day with a minimum of 2 hours apart, following 1 hour class and also distracting activities during the interval between administrations. Both versions were administered in individual random order. Given the bilingual characteristics of the study population also the language (Spanish or Catalan) was individually randomized, and each student answered the web and paper version of the same language. The fieldwork was carried out between October and November 2013.

### Ethics statement

All students whose parents signed consent to participate and they voluntarily agreed to participate were included in the study. All procedures were carried out following the data protection requirements of the European Parliament (Directive 95/46/EC of the European Parliament and of the Council of 24 October 1995 on the protection of individuals with regard to the processing of personal data and on the free movement of such data). The ethical and legal requirements in Spain were also adhered to, and the protocol was approved by the Clinical Research Ethical Committee of the Hospital del Mar, Barcelona.

### Measures

The KIDSCREEN-52 is a self-reported, generic measure of HRQOL for use in children and adolescents [6]. The KIDSCREEN-52 measures HRQOL in ten dimensions: Physical Well-being (PH, five items); Psychological Wellbeing (PW, six items); Moods and Emotions (ME, seven items); Self-perception (SP, five items); Autonomy (AU, five items); Parent Relation and Home Life (PA, six items); Social Support and Peers (PE, six items); School Environment (SC, six items); Social Acceptance (bullying) (BU, three items); Financial Resources (FI, three items). The KIDSCREEN-52 item uses five-point Likert-type scales to assess either the frequency (never, seldom, sometimes, often, always) or intensity (not at all, slightly, moderately, very, extremely). The recall period is 1 week. Scores for each dimension are calculated using Rasch analysis and then transformed into T-values with a mean of 50 and a standard deviation (SD) of 10. Higher scores indicate better HRQOL. KIDSCREEN-52 T scores refer to the mean values and SD from a representative sample of the European general population [10]. The Spanish and Catalan paper versions of the KIDSCREEN-52 showed acceptable levels of reliability as well as construct and convergent validity. Cronbach's alpha values ranged from 0.74 to 0.86 for the different dimensions, and intraclass correlation coefficients ranged from 0.55 to 0.79. It fulfills requirements of the Rasch model in showing no differential item functioning by age group or sex [11]. As the KIDSCREEN-52 was only designed to give dimension scores, not an overall score, we also calculated KIDSCREEN-10 scores. The KIDSCREEN-10 was specifically designed to be used as an index score and meets all of the necessary criteria, including unidimensionality, good internal consistency (0.82), and satisfactory construct, concurrent and discriminant validity [12].

Other variables collected in the present study included age, sex, family socio-economic status, family type, and parental level of education. Socio-economic status was measured using the Family Affluence Scale (FAS) [13], which includes family car ownership, having their own unshared room, the number of computers at home, and how many times they spent on holidays in the past 12 months. FAS scores were categorized as low (0–3), intermediate (4–5), and high (6–7) affluence level. Sociodemographic information collected from parents included the highest family level of education categorized as primary education, secondary education, and university degree.

Children's mental health status was assessed using the Strengths and Difficulties Questionnaire (SDQ). The SDQ is a brief behavioural screening questionnaire for children and adolescents that asks about their mental health symptoms and positive attitudes [14]. The instrument consists of 25 items measuring 5 dimensions. Higher scores indicate more problems except on the pro-social behaviour dimension. Items in the 4 problem dimensions are summed to give a total difficulties score ranging from 0 (no problems) – 40 (maximum problems). The Spanish version has been shown to be reliable and valid [15].

### Statistical analysis

Mean HRQOL scores were compared between web and paper versions using paired T Test and standardized mean differences were computed (effect size, ES) to evaluate the degree of differences. ES lower than 0.2 was considered as absence of differences, 0.21 to 0.5 as low, 0.51 to 0.8 as moderate and >0.8 as high [16]. It was expected that the Spanish and Catalan eKIDSCREEN would present similar scores to the paper version with no differences greater than 0.2 standard deviation (SD). Missing values, and floor and ceiling effect were estimated expecting less than 15% in all dimensions. The internal consistency of the KIDSCREEN-52 dimensions was calculated using Cronbach's alpha [17]. Alpha coefficients of 0.7 or higher were considered acceptable.

Agreement was analyzed through Intraclass correlation coefficients (ICC) [18], Bland and Altman plots, and 95% Confidence Interval (95% CI) was also calculated for the upper and lower limits of agreement [19]. An ICC lower than 0.4 was considered as very low, 0.4 to 0.74 as low to acceptable, and 0.75 or higher as excellent [20]. It was expected that the psychometric properties of the digital version would be similar to the paper version: it was expected similar floor and ceiling effects, all dimensions would show an internal consistency >0.7, agreement with the paper version would be acceptable (ICC at least 0.4 to 0.74). Additionally, mean score comparison was also carried out according to the language and order of administration.

Construct validity was assessed analyzing the KIDSCREEN-52 dimension scores following the known groups approach according to age, gender, and the SDQ total difficulties stratified by unlikely vs probable/possible case. A priori hypotheses about the relative magnitude of the differences were specified according to previous studies [6], [10], [11], and ES was calculated to estimate the magnitude of differences comparing web and paper versions. It was expected that the web version would present similar results in terms of construct validity than the paper version (according to age, sex, socioeconomic status, and mental health status).

All statistical analyses were repeated for the total sample and stratified by order of administration and language.

## Results

Minor changes have been made to the internet version after the pilot test. Participation rate was 77% (n = 715). 54% of the sample were girls, mean age was 11.7y; 23% came from families with university degree, and 30% were in the low FAS (table 1).

**Table 1:** Sociodemographic characteristics of the 713 participants in a comparison of digital and paper versions of the Spanish and Catalan versions of the KIDSCREEN-52.

	N	Mean or %
Age		
Mean (SD)	713	11.7y (2.8)
7–11	214	30.7
12–15	446	64.0
≧16	37	5.3
Sex		
Female	385	54.0
Male	328	46.0
Family type		
Biparental	573	84.0
Monoparental	109	16.0
Highest family level of education		
Primary education	163	25.1
Secondary	554	51.4
University degree	153	23.5
Family Affluence scale		
Low	214	30.7
Middle	446	64.0
High	37	5.3
Order of administration		
Web version first	338	47.4
Paper version first	375	52.6

SD: standard deviation. Missing value: age (2); sex (2); type of family (33); family affluence scale (18); level of education (65); order of administration (2).

Mean score comparisons between web and paper versions of the KIDSCREEN-52 and the KIDSCREEN-10 Index showed no differences, except for the dimension of Peers and social support (p = 0.04) (table 2). ES was lower than 0.2 in all dimensions. Percentage of missing values was slightly lower in the web version compared to the paper version (web version n = 2; paper version ranged from n = 7 in BU to n = 30 in the KS-10 Index). Ceiling effect was higher than 15% in 9 out of ten dimensions, and similar in both web and paper versions. The KIDSCREEN-10 Index showed low percentage of floor effect and no ceiling effect in both versions. Internal consistency in the web version (Cronbach's alpha ranged 0.7 to 0.88) was also similar to the paper version.

**Table 2:** Comparison of scores, floor and ceiling effect, and internal consistency coefficients between the web and paper versions of the KIDSCREEN (n = 682–713), Palafolls study, 2013.

	Mean score paper version (SD)	Mean score web version (SD)	Paired T-test *T value*	ES	Floor effect (web) (%)	Ceiling effect (web) (%)	Floor effect (paper) (%)	Ceiling effect (paper) (%)	Cronbach alpha (web version)	Cronbach alpha (paper version)
PHY	53.9 (11.6)	53.9 (11.6)	0.96	0.002	0.0	14.4	1.7	15.1	0.81	0.83
PSY	55.3 (10.2)	55.6 (10.7)	0.44	0.002	0.3	27.2	2.2	24.5	0.87	0.86
ME	52.3 (11.8)	52.0 (12.3)	0.55	0.02	0.6	19.1	0.3	18.2	0.88	0.87
AU	52.2 (11.2)	52.0 (11.2)	0.43	0.02	0.6	19.1	0.4	19.8	0.86	0.70
SP	52.3 (10.6)	52.0 (10.6)	0.46	0.01	0.1	19.1	1.7	19.1	0.70	0.87
PA	54.2 (9.4)	54.1 (10.0)	0.68	0.009	0.1	30.3	0.0	28.1	0.85	0.82
PE	54.6 (10.1)	55.2 (10.4)	***0.04***	0.05	0.1	19.4	0.1	17.4	0.82	0.78
SC	53.5 (12.4)	52.2 (12.6)	0.24	0.02	0.7	16.4	0.6	16.0	0.88	0.87
BU	48.0 (11.2)	47.5 (11.8)	0.15	0.04	1.7	44.0	0.7	44.3	0.78	0.75
FI	49.8 (9.2)	49.6 (9.3)	0.41	0.02	1.8	20.3	1.8	19.9	0.83	0.84
KS-10 index	54.6 (11.9)	54.4 (11.7)	0.55	0.01	5.6	0.0	6.3	0.0	0.80	0.81

SD: standard deviation; ES: Standardized mean differences (effect size). Missing values: web version (2); paper version (ranged from n = 7 for BU to n = 30 for the KDS-10 Index). PHY Physical well-being; PSY: Psychological well-being; ME: Moods and emotions; AU: Autonomy: SP: Self-perception; PA: Parent Relation and Home Life; PE: Social Support and Peers; SC: School Environment; BU: Social Acceptance (bullying); FI: Financial Resources; KS-10 Index: KIDSCREEN-10 Index.

The degree of agreement of web and paper versions was excellent (ICC range = 0.75 to 0.87) (table 3). Lower and upper limits of agreement showed relatively high variability.

**Table 3:** Level of agreement of web and paper versions of the KIDSCREEN-52 (n = 682–713). Palafolls study, 2013.

	ICC (95%CI)	95% CI lower agreement limit	95% CI upper agreement limit
PHY	0.80 (0.77–0.82)	−14.07/−13.55	13.56/15.58
PSY	0.76 (0.73–0.79)	−15.07/−14.03	13.62/14.66
ME	0.77 (0.74–0.80)	−16.67/−15.46	15.83/17.04
AU	0.78 (0.75–0.80)	−14.18/−13.15	13.56/14.59
SP	0.80 (0.78–0.83)	−14.42/−13.37	13.76/14.81
PA	0.81 (0.79–0.84)	−12.23/−11.37	11.20/12.06
PE	0.75 (0.72–0.78)	−15.37/−14.3	13.22/14.29
SC	0.87 (0.85–0.89)	−12.80/−11.87	12.42/13.35
BU	0.70 (0.66–0.73)	−17.81/−16.50	17.45/18.76
FI	0.80 (0.77–0.83)	−11.82/−10.97	11.32/12.17
KS-10 index	0.80 (0.77–0.82)	−15.37/−14.24	14.61/15.74

95%CI: 95% confidence interval; ICC: intraclass correlation coefficient. PHY Physical well-being; PSY: Psychological well-being; ME: Moods and emotions; AU: Autonomy: SP: Self-perception; PA: Parent Relation and Home Life; PE: Social Support and Peers; SC: School Environment; BU: Social Acceptance (bullying); FI: Financial Resources; KS-10 Index: KIDSCREEN-10 Index.

**Figure 1:**
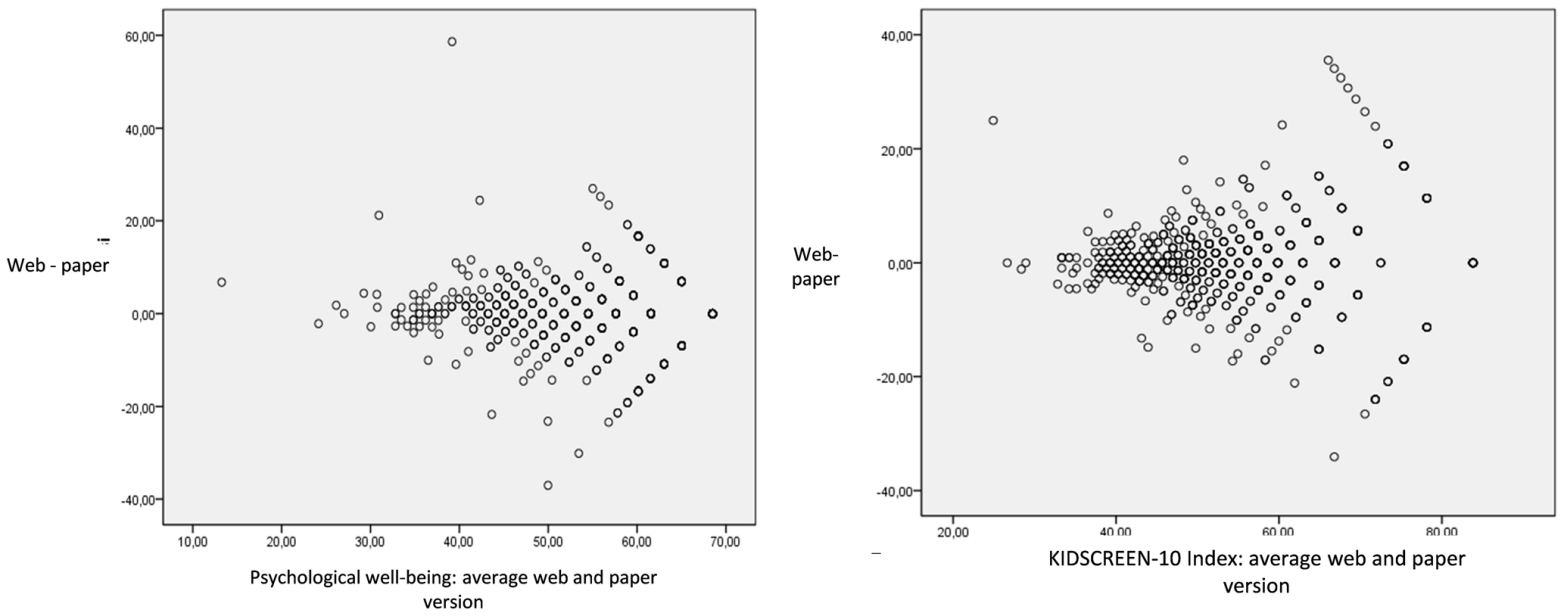
Bland and Altman plots of Psychological well-being and the KIDSCREEN-10 Index.

The level of agreement varied according to the HRQOL level (Figure 1). Agreement was better for worst values in almost all dimensions and the KIDSCREEN-10 Index.

Table 4 shows score comparisons according to the language version (Catalan or Spanish) and the order of administration (web or paper version first). ES lower than 0.2 were seen in case of language comparisons and minimal differences were found on Physical well-being (ES = 0.2) and Moods and Emotion (ES = 0.25) when the web version was administered after the paper version. Internal consistency was also similar comparing both language versions and the order of administration (data not shown).

**Table 4:** Comparison of scores according to the language (Spanish or Catalan) and order of administration (web or paper first) first (n = 682–713), Palafolls study, 2013.

	Catalan versión (n = 360) (SD)	Spanish versión (n = 353) (SD)	T test	ES	First web version (n = 338) (SD)	2nd web version (n = 375) (SD)	T test	ES
PHY	53.7 (11.6)	54.7 (11.5)	NS	0.08	52.7 (10.7)	55.0 (12.2)	***0.008***	0.2
PSY	54.9 (10.6)	56.2 (10.8)	NS	0.12	55.0 (9.5)	56.0 (11.5)	NS	0.09
ME	51.5 (12.4)	52.5 (12.2)	NS	0.08	50.4 (11.5)	53.5 (12.9)	***0.001***	0.25
AU	51.7 (11.4)	52.2 (10.9)	NS	0.04	51.3 (10.6)	52.5 (10.6)	NS	0.11
SP	51.2 (10.3)	52.9 (10.9)	***0.03***	0.16	51.9 (10.6)	52.1 (10.7)	NS	0.01
PA	53.6 (9.9)	54.6 (10.0)	NS	0.10	54.3 (9.0)	54.0 (10.8)	NS	0.03
PE	54.4 (10.7)	56.0 (10.0)	***0.03***	0.15	55.4 (9.7)	55.0 (11.0)	NS	0.03
SC	52.7 (12.5)	53.7 (12.8)	NS	0.07	53.3 (11.9)	53.1 (13.2)	NS	0.01
BU	46.6 (12.2)	48.4 (11.4)	***0.03***	0.15	46.7 (11.6)	48.2 (11.9)	NS	0.03
FI	49.3 (9.3)	49.8 (9.3)	NS	0.05	49.8 (8.5)	49.4 (10.0)	NS	0.03
KS-10 index	53.8 (11.3)	55.0 (12.2)	NS	0.10	53.7 (10.9)	55.0 (12.3)	NS	0.01

SD: standard deviation; ES: Standardized mean differences (effect size). Missing values: web version (2); paper version (ranged from n = 7 for BU to n = 30 for the KDS-10 Index). PHY Physical well-being; PSY: Psychological well-being; ME: Moods and emotions; AU: Autonomy: SP: Self-perception; PA: Parent Relation and Home Life; PE: Social Support and Peers; SC: School Environment; BU: Social Acceptance (bullying); FI: Financial Resources.

Results from the web version showed that boys scored higher than girls with statistically significant differences in all dimensions (p<0.01) except on SC, BU and FI; younger children scored better than older adolescents in all dimensions except on BU and FI; statistically significant differences were also found according to the FAS with worse scores in all dimensions in those children reporting low family affluence. Similar results were found with the paper version (data not shown and available from authors upon request). Table 5 shows KIDSCREEN scores according to mental health status. Probable/possible cases show lower (worse) scores in all dimensions, with higher ES in the dimensions of Moods and Emotions (ES = 0.98 in both versions) and Psychological well-being (ES = 0.78 and 0.86 for web and paper versions, respectively).

**Table 5:** Comparison of the web and paper versions of the KIDSCREEN by mental health status according to the Strengths and Difficulties Questionnaire (SDQ, probable case), Palafolls study, 2013 (n = 682–713).

	Web version			Paper version		
	Unlikely Mean (SD)	Probable/possible case Mean (SD)	Effect size	Unlikely Mean (SD)	Probable/possible case Mean (SD)	Effect size
PHY	54.9 (11.1)	48.5 (13.0)	0.55	54.5 (11.1)	50.0 (14.2)	0.38
PSY	56.6 (9.7)	48.5 (13.4)	0.78	56.4 (9.4)	47.8 (12.6)	0.86
ME	53.8 (11.3)	42.3 (13.4)	0.98	53.9 (10.9)	43.0 (11.9)	0.98
AU	52.8 (10.6)	46.6 (13.4)	0.55	52.9 (10.6)	47.0 (13.6)	0.52
SP	53.0 (10.3)	45.7 (10.6)	0.70	53.1 (10.2)	46.3 (11.1)	0.65
PA	55.1 (9.3)	48.3 (11.6)	0.69	55.0 (9.1)	49.3 (10.0)	0.60
PE	55.9 (10.0)	50 (11.6)	0.49	55.1 (9.6)	51.7 (12.1)	0.32
SC	54.1 (12.1)	46.7 (14.3)	0.59	54.3 (11.7)	46.5 (14.3)	0.64
BU	49.0 (10.5)	38.9 (14.7)	0.78	49.2 (10.3)	40.7 (13.1)	0.78
FI	50.2 (9.1)	45.9 (10.3)	0.46	50.3 (8.8)	46.9 (10.6)	0.36
KS-10 index	55.8 (11.3)	46.1 (10.6)	0.85	55.7 (11.5)	46.6 (10.9)	0.79

PHY Physical well-being; PSY: Psychological well-being; ME: Moods and emotions; AU: Autonomy: SP: Self-perception; PA: Parent Relation and Home Life; PE: Social Support and Peers; SC: School Environment; BU: Social Acceptance (bullying); FI: Financial Resources; KS-10 Index: KIDSCREEN-10 Index.

No differences were found stratifying the sample according to the language. Slightly higher (better) score, although without statistically significant differences, were seen when web version was administrated after the paper version (data not shown and available from authors upon request).

## Discussion

The present study demonstrates that both web and paper versions showed comparable scale scores, and psychometric properties, and also an excellent level of agreement. The results of the study support that web-based Spanish version of the KIDSCREEN-52 is feasible, and as reliable and valid as the paper version.

Some studies have demonstrated that online health questionnaires are feasible especially among adolescents [21], and showed similar scores and psychometric properties compared to the paper version [22]. A computer-based questionnaire offers a number of advantages and limitations comparing with paper-and-pencil versions. Missing values or incomplete data can be reduced by requiring completion of an item before the individual can move on to the next question, and out-of-range values can be eliminated. Personnel time can be saved by reducing the amount of time spent entering data and handling paper, which also increases the accuracy of data by reducing typing errors. In addition, computer software scores the patient responses immediately and creates summary information. A meta-analytic review concluded that extensive evidence indicates that paper and computer versions of self-reported questionnaires are equivalent [3]. Nevertheless, some differences between the web and paper version exist. In the paper version all items are visible all the time, while usually only one item or a set of items is visible at a time in the computer. When completing the paper version, one can create an overall impression of the questionnaire before choosing the appropriate option for each item. In addition, the paper version allows the opportunity to change/correct one of the responses if the subject so chooses. In the computer version, it is not possible to go back to change any previous answers. On the other hand, the subject must respond to each set of questions as the software used in the study did not allow the subject to move on without answering each set of questions.

The potential usefulness of measures of child HRQOL using ICTs are in the evaluation of the effectiveness of health interventions, and in daily clinical practice. One barrier to implement these measures in clinical practice is to find time and space for patients to answer the questionnaires before the consultation, the subsequent entry into database, and the need for statistical software to implement algorithms complex scoring. Instead digital versions are a good way to solve most of these barriers.

In the present study the psychometric properties were similar between the two modes of administration. Both versions showed a similar high ceiling effect and acceptable internal consistency. Both versions also showed a similar ability to discriminate between known groups and according to mental health status. One of the strengths of the study was that all participants answered both versions and also that both versions were randomly administered.

Some limitations of the study deserve comments. First, the sample selected in the present study to compare the web-based and paper versions included the whole school population of the village of Palafolls that is not necessarily representative of the Spanish and Catalan childhood population. Nevertheless, in this type of studies it is not necessary for the samples to be representative, but they should include a wide range of possible answers. This ensures and facilitates comparisons within the sample. Secondly, the fact that both versions have been administered on a single day may have caused some recall. Nevertheless, no clear evidence exists on the ideal time span for gathering test-retest reliability. According to some authors it could be 2 weeks [23]. A study carried out in adult population on test-retest reliability of health status instruments using 2-day or 2-week time frame between administrations did not affect the results of the reliability testing [24]. Another study in children using similar strategy than the present work, administering the questionnaires the same day with distracting activities before starting the second mode showed acceptable results [25]. A certain retest effect has been described with improvements in the second administration independently of the time between measures [26]. In the present study the order of completing web and paper versions was randomized and scores were slightly better when the web version was administered after the paper version. Nevertheless, these differences were minimal in terms of ES and only in two dimensions of the KIDSCREEN (ES = 0.2 and 0.25 respectively). These results reinforce the idea that, although there was some memory effect, this effect did not show an important impact on the level of agreement between the web and paper versions. Future studies could add more information on the appropriate time-interval for scores comparisons by administering the two versions within two hours at a later time-point (i.e. two weeks or two months apart). A matter of future studies would also be the use of techniques such as the item response theory (IRT) [27]. IRT might be useful to identify potential predictors of discrepancies at item level, i.e. items functioning differently for respondents from different languages or according to the order of administration”. Thirdly, the statistical methods used in this study have strengths and weaknesses. The ICC combines information about bias and association as it reflects both the degree of correspondence and agreement between ratings, takes account of the actual magnitude of the score and is sensitive to systematic bias in data. One disadvantage is that ICC is strongly influenced by the variance. The Bland and Altman method has advantages compared with ICC, such as easily-detected visual representation of the degree of agreement, easy identification of bias outliers, and easy detection of any relationship between variability of measure with the size of mean. The major disadvantages of Bland and Altman method are the complexity of interpretation compared with a single reliability index, and the need for a sample set over 50 subjects to avoid very wide 95%CI for limits of agreement. Finally, the average time to complete the web version was 9.15 minutes, and no comparable data were possible to collect with the paper version.

A promising area of research in which KIDSCREEN electronic is contributing to is in the development of the KIDS-CAT [28], a computer-adaptive test that is in the process of development in German speaking countries. They apply analogous system to the methods used by the US-wide patient-reported outcome initiative Patient Reported Outcomes Measurement Information System (PROMIS) [29]. The KIDS-CAT content is based on the KIDSCREEN-27 domain structure, and item banks including all KIDSCREEN items plus items used in other established pediatric health instruments. The development of the Spanish eKIDSCREEN allows to establish some comparisons with these instruments.

In summary, the results show that the eKIDSCREEN is equally reliable and valid than the paper version to be administered in children and adolescents. It will incorporate the child population in the assessment of HRQOL using a medium in which they are handled flexibly and autonomously, providing a more attractive format, and easier to manage and analyze than the paper versions.
