# Correction: Comparison of the Web-Based and Digital Questionnaires of the Spanish and Catalan Versions of the KIDSCREEN-52

**DOI:** 10.1371/journal.pone.0116619

**Published:** 2015-01-26

**Authors:** The PLOS ONE Staff

There is an error in the article title. The word “Digital” should read “Paper.” The correct title is: Comparison of the Web-Based and Paper Questionnaires of the Spanish and Catalan Versions of the KIDSCREEN-52. The correct citation is: Rajmil L, Robles N, Rodriguez-Arjona D, Azuara M, Codina F, et al. (2014) Comparison of the Web-Based and Paper Questionnaires of the Spanish and Catalan Versions of the KIDSCREEN-52. PLoS ONE 9(12): e114527. doi:10.1371/journal.pone.0114527

Additionally, there is a missing affiliation for author Noemí Robles. Noemí Robles is also affiliated with: Red de Investigación en Servicios de Salud en Enfermedades Crónicas (REDISSEC), Barcelona, Spain.
